# Temporal Changes and Between-Competition Differences in the Activity Profile of Elite Hurling Referees

**DOI:** 10.3390/jfmk9040271

**Published:** 2024-12-13

**Authors:** Aidan J. Brady, Michael Scriney, Mark Roantree, Andrew McCarren, Niall M. Moyna

**Affiliations:** 1 School of Health and Human Performance, Dublin City University, D09 YH9P Dublin, Ireland; 2 Insight Centre for Data Analytics, Dublin City University, D09 V209 Dublin, Ireland; 3 School of Computing, Dublin City University, D09 V209 Dublin, Ireland

**Keywords:** decision-making, GPS, invasion field-based team sport, running demands, time–motion analysis

## Abstract

**Background/Objectives:** This study examined the activity profile of elite hurling referees during games in the National Hurling League (NHL) and All-Ireland Championship (AIC) and across all divisions of the NHL and phases of the AIC. Temporal changes between the first and second half and across the four quarters were also examined. **Methods:** Data were collected from 36 referees using 10-Hz global positioning system technology during 106 NHL and 85 AIC games and analyzed for duration, total distance, very low-speed movement (<0.69 m·s^−1^), walking (≥0.69–1.59 m·s^−1^), low-speed running (≥1.60–3.08 m·s^−1^), moderate-speed running (≥3.09–4.62 m·s^−1^), high-speed running (HSR) (≥4.63–6.34 m·s^−1^), and very high-speed running (VHSR) (≥6.35 m·s^−1^) distance and peak running speed. **Results:** Total distance was not significantly different between games in the NHL (120.7 ± 9.4 m·min^−1^) and AIC (122.8 ± 9.9 m·min^−1^, ES = 0.04). There was also no significant difference in HSR distance between the NHL (17.1 ± 6.5 m·min^−1^) and AIC (18.0 ± 7.2 m·min^−1^, ES = 0.10). The only significant difference between the NHL and AIC was in LSR distance, which was higher in the AIC (ES = 0.24). VHSR distance was significantly higher during games in Division 3A+3B (0.7 ± 0.8 m·min^−1^) compared to Division 1A+1B (0.2 ± 0.3 m·min^−1^) and Division 2A+2B (0.1 ± 0.3 m·min^−1^). HSR distance was significantly lower during games in the All-Ireland Series (15.7 ± 6.7 m·min^−1^) compared to games in the Leinster (21.1 ± 8.5 m·min^−1^) and Munster (17.9 ± 5.4 m·min^−1^) Championships. **Conclusions:** The findings of this study can be used to support the development of physical conditioning programs that are specific to the demands experienced during competitive match play.

## 1. Introduction

Hurling is a stick and ball, invasion field-based team sport (IFTS). Games are played between two teams comprising 15 players each, on a playing area measuring approximately 130–145 m × 80–90 m [1]. Hurling involves brief periods of high-speed activity interspersed with low-speed activity, performed over two 35 min halves. The periods of high-speed activity are unpredictable, influenced by the patterns of play and can vary greatly from one game to another [1]. Similar to other IFTSs like Gaelic football [2] and soccer [3], hurling is officiated by one referee who is assisted by two side-line officials. There are also four goal line umpires, one at each goal post, who are responsible for adjudicating the outcome when the ball crosses the end line. While several studies have examined the activity profile of elite hurling players, reporting total distances of ~109 m·min^−1^, high-speed running distances of ~16.7 m·min^−1^ (≥4.72 m·s^−1^), and peak running speeds of ~8.5 m·s^−1^ [4,5,6], no studies have examined the activity profile of elite hurling referees. The design of physical conditioning programs has consequently been based on existing analyses of the physical demands of refereeing during other IFTSs, which may not adequately prepare hurling referees for the demands of match play.

Competitions in hurling are organized at both sub-elite club level and elite inter-county level [7]. Inter-county teams comprise the top-rated club players within each county. The National Hurling League (NHL) and the All-Ireland Championship (AIC) are the two major inter-county competitions contested each year. The NHL, which typically commences in January, involves six divisions of six teams, except the bottom division, which has five teams that are divided based on ranking. Each team plays each other once, either home or away. The AIC is regarded as the premier inter-county competition and succeeds the NHL each year [1]. The AIC begins with the provincial championships (Leinster and Munster) before progressing to the All-Ireland Series (AIS), which involves the quarter-finals, semi-finals, and final.

Comparison of the activity profile of elite players between the NHL and AIC found that players cover a greater relative and sprint distance (≥6.11 m·s^−1^) and have a higher peak running speed during games in the AIC than the NHL. Elite players also perform fewer sprints during the NHL than the AIC [4]. These differences may have important implications for the preparation of hurling referees given the relation of the activity profile of IFTS players and referees [8]. By contrast, in Gaelic football, a native Irish sport that shares a number of similarities with hurling including pitch size, player numbers, and game duration, no differences were found in the activity profile of elite referees between the national league and AIC [9], but no comparison between competitions has been performed for elite Gaelic football players.

Alongside the analysis of the full game, the examination of temporal changes in the activity profile provides useful information on the differences between match halves and quarters that can be incorporated into physical conditioning programs. Several studies investigating temporal changes in the activity profile of elite IFTS referees have reported differences in activity between halves and/or between quarters [2,10,11]. In Gaelic football, elite referees cover a greater total and high-speed distance (≥4.87 m·s^−1^) during the first half, which is largely attributed to the intense first quarter, during which referees total and high-speed running (HSR) distance is higher than any other quarter of match play [2]. In soccer, temporal analysis of refereeing performance found that the distance of the referee from decisions [12] and the number of incorrect decisions [13] increases in the second half of games, highlighting the importance of physical conditioning for referees.

The aims of this study were (i) to compare the activity profile during games in the NHL and AIC, (ii) to examine temporal changes in the activity profile during games in the NHL and AIC, and (iii) to compare the activity profile between the divisions of the NHL and phases of the AIC. It was hypothesized that elite hurling referees would (i) cover a greater relative distance and distance at high speed during games in the AIC than in the NHL, (ii) cover a greater relative distance and distance at high speed during the first quarter compared to any other quarter of match play during games in both the NHL and AIC, and (iii) cover a greater relative distance and distance at high speed during games in the latter stages of the AIS than in the provincial championships.

## 2. Materials and Methods

The activity profile of elite hurling referees was examined using global positioning system (GPS) technology during competitive match play across four competitive seasons. Participants were recruited from the Gaelic Athletic Association (GAA) senior inter-county panel, which is selected prior to the start of the NHL and AIC each year. The dataset comprises games from all divisions of the NHL and all phases of the AIC including quarter-finals, semi-finals, and finals. Activity data from the GPS units were analyzed for game duration, total distance, distance covered in six distinct movement categories, and peak running speed. Activity data were also separated into halves and quarters for temporal analyses.

Thirty-six elite inter-county hurling referees (mean ± SD; age, 39.7 ± 5.5 yr; height, 1.77 ± 0.06 m; body mass, 84.2 ± 10.0 kg; experience at national level, 6.9 ± 5.4 yr) gave written informed consent to participate after written and verbal explanations of the study procedures. Ethical approval was obtained from Dublin City University Research Ethics Committee (permit: DCUREC/2018/041) in accordance with the declaration of Helsinki, with the exception of preregistration in a trial database. A total of 191 full game datasets were collected. All participants were selected for the elite GAA senior inter-county referee panel during the data collection period, which is chosen on an annual basis prior to the start of each competition.

Referees wore 10-Hz GPS units (STATSports, Newry, Ireland) midway between the scapulae in a custom-made undergarment during match play. GPS units were activated a minimum of 15 min prior to the start of each game to establish a satellite lock [14]. These units have a mean bias of 1.1–2.0% for distance covered during a 400 m lap of an athletics track, 2.3–2.7% for the distance covered during a 128.5 m team sport circuit, 1.1–1.3% for the distance covered during a 20 m run, and 1.8–2.4% for peak running speed [15,16]. The inter- and intra-unit reliability for distance covered across different movement circuits and at different speeds, including those exceeding 5 m·s^−1^, and the inter-unit reliability for peak running speed is also good (CV < 5%) [17,18]. Data from the GPS units were downloaded to the manufacturer’s software and separated into first and second halves, including the additional time at the end of each half and excluding the warm-up and half-time period. Raw data were exported and analyzed further using the Python (v.3.7) programming language (Python Software Foundation, Wilmington, DE, USA).

Data points in each full game dataset were excluded from the analysis if instantaneous velocity was >10 m·s^−1^, instantaneous acceleration was >6 m·s^−2^ or instantaneous deceleration was >6 m·s^−2^ [19,20]. Datasets were also examined for horizontal dilution of precision (HDOP) and the number of satellites locked to the unit. Individual data points were excluded if the HDOP was >2.0 or if fewer than 8 satellites were locked to the unit [21]. When a data point was excluded, the instantaneous velocity was imputed using the median of the preceding and successive data points. For the 191 full game datasets, an average of 71 ± 147 (range: 0–792) individual data points were excluded, corresponding to 0.2 ± 0.3% of the data (range: 0.0–1.8%). No datasets exceeded the a priori threshold of >3% for excluded data. The 191 full game datasets comprised 106 NHL games (Division 1A+1B, n = 58; Division 2A+2B, n = 33; Division 3A+3B, n = 15) and 85 AIC games (Leinster, n = 26; Munster, n = 25; AIS, n = 34). The median number of full game datasets per referee was 4, with a mean of 5.3 and range of 1–16 games.

The movement variables analyzed were total distance, distance covered in the very low-speed movement (VLSM) (<0.69 m·s^−1^), walking (≥0.69–1.59 m·s^−1^), low-speed running (LSR) (≥1.60–3.08 m·s^−1^), moderate-speed running (MSR) (≥3.09–4.62 m·s^−1^), HSR (≥4.63–6.34 m·s^−1^), and very high-speed running (VHSR) (≥6.35 m·s^−1^) categories, and peak running speed. The boundaries of the movement categories were developed specifically for the analysis of elite hurling referees [19]. Total distance and the distance covered in each movement category was reported in relative terms, expressed as meters and distance per unit time (m·min^−1^). To examine temporal changes in the activity profile, full game datasets were divided into first and second halves, and into four quarters, each representing the first and last fifteen minutes of each half.

Data are presented as mean ± SD, unless otherwise stated. Temporal changes in the movement variables were examined using a series of linear mixed models (LMM). Match period was treated as a repeated measure with competition phase and playing season included as fixed effects. Referee and a referee-match period interaction were treated as random effects. The covariance structure for each model was determined using the Akaike Information Criterion. For relative distance and distance in each movement category, except for distance covered in the VHSR category, a compound symmetry heterogenous structure was fitted. An unstructured covariance structure was fitted for VHSR distance and peak running speed. Normality assumptions of the residuals were examined using plots of the model residuals and the predicted values. Distance covered in the VLSM, HSR, and VHSR categories violated these assumptions and were log transformed. Significant main effects and interactions were probed using Bonferroni adjusted contrasts. Estimates of effect size (ES) were determined using the mean difference and the square root of the residual components for each model [22], providing an estimate analogous to Cohen’s d. The significance level was set at α ≤ 0.05 for all tests.

## 3. Results

During the NHL and AIC, the mean game duration was 76.8 ± 2.2 min and 77.0 ± 2.0 min, during which referees covered a relative distance of 120.7 ± 9.4 m·min^−1^ and 122.8 ± 9.9 m·min^−1^, respectively. There was no significant difference in the mean game duration (*p* = 0.067, ES = 0.26) or relative distance (*p* = 0.750, ES = 0.04) between the NHL and AIC. The relative distance in each movement category during the NHL and AIC is presented in Table 1. The peak running speed was not significantly different (*p* = 0.834, ES = 0.03) between the NHL (6.59 ± 0.46 m·s^−1^) and the AIC (6.60 ± 0.37 m·s^−1^).

The duration of the first and second half was 37.7 ± 1.4 min and 39.1 ± 1.5 min (*p* < 0.001, ES = 1.00), respectively, in the NHL. In the AIC, the duration of the first and second half was 37.7 ± 1.2 min and 39.3 ± 1.6 min (*p* < 0.001, ES = 1.15), respectively. There was no significant difference in the duration of the first (*p* = 0.266, ES = 0.19) or second half (*p* = 0.475, ES = 0.23) between the NHL and AIC. The relative distance in the first and second half was 124.1 ± 10.1 m·min^−1^ and 117.5 ± 10.3 m·min^−1^ (*p* < 0.001, ES = 0.57), respectively, in the NHL. In the AIC, the relative distance in the first and second half was 126.0 ± 11.1 m·min^−1^ and 119.9 ± 10.3 m·min^−1^ (*p* < 0.001, ES = 0.53). There was no significant difference in the relative distance of the first (*p* = 0.647, ES = 0.07) or second half (*p* = 0.883, ES = 0.02) between the NHL and AIC. The relative distance in each movement category during the first and second half of the NHL and AIC is presented in Table 2. There were no significant differences in the relative distance in each movement category between the NHL and AIC during the first and second half. Peak running speed did not differ between the first and second half of the NHL (*p* = 799, ES = 0.02) or the AIC (*p* = 0.604, ES = 0.05).

**Figure 1:**
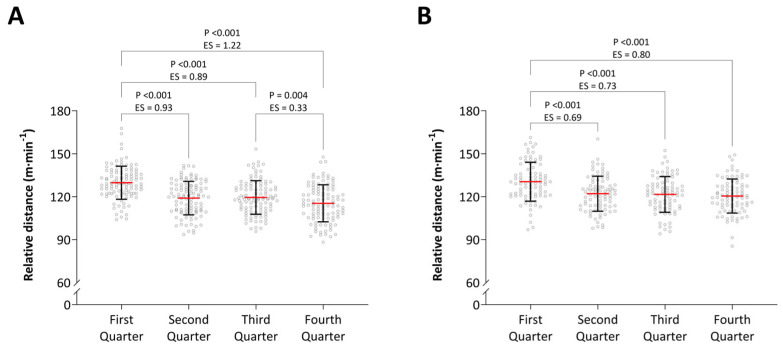
Distance across each quarter of match play in the NHL (**A**) and AIC (**B**). Data are presented as mean values with error bars representing SD and circles representing individual data points.

**Figure 2:**
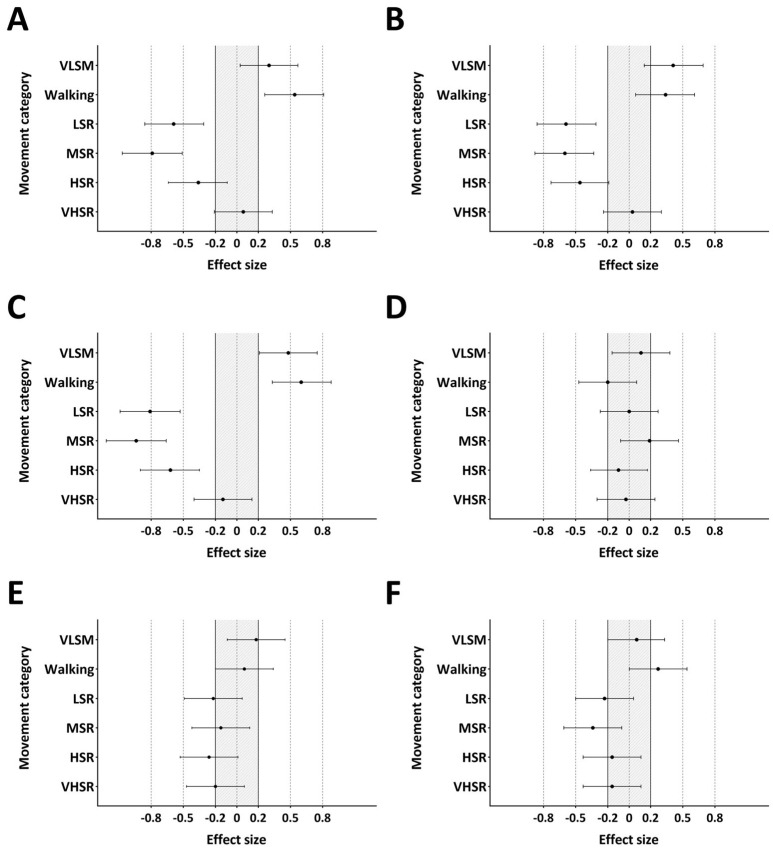
Effect size plots of the change in distance in each movement category between the first and second quarter (**A**), first and third quarter (**B**), first and fourth quarter (**C**), second and third quarter (**D**), second and fourth quarter (**E**), and third and fourth quarter (**F**) of match play in the NHL. Circles represent the ES, and the horizontal lines represent the 95% confidence interval. The grey shaded area represents trivial ES. VLSM, very low-speed movement; LSR, low-speed running; MSR, moderate-speed running; HSR, high-speed running; VHSR, very high-speed running.

**Figure 3:**
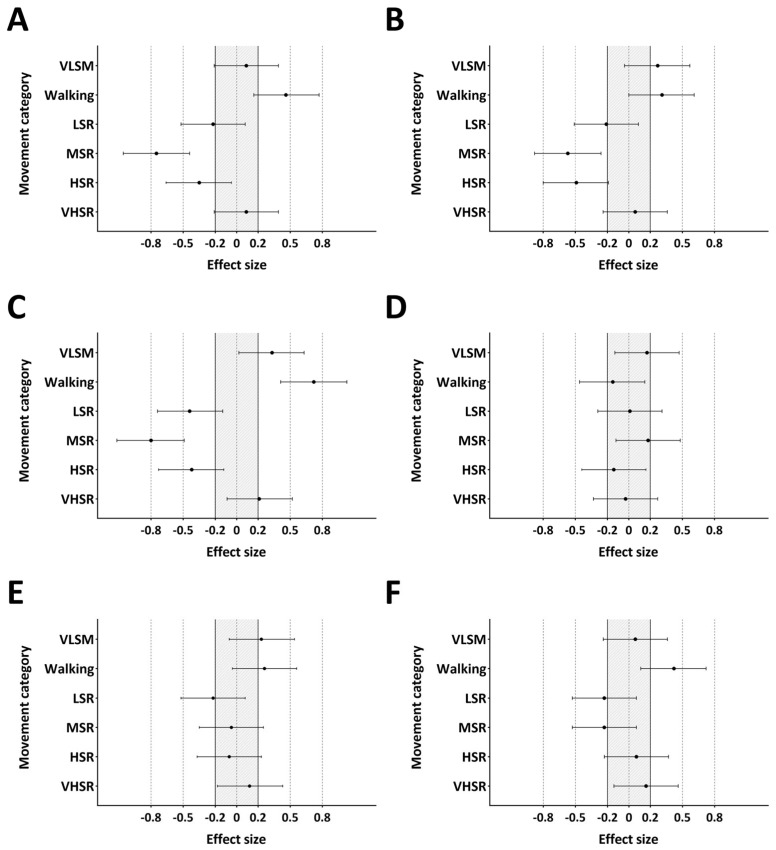
Effect size plots of the change in distance in each movement category between the first and second quarter (**A**), first and third quarter (**B**), first and fourth quarter (**C**), second and third quarter (**D**), second and fourth quarter (**E**), and third and fourth quarter (**F**) of match play in the AIC. Circles represent the ES, and the horizontal lines represent the 95% confidence interval. The grey shaded area represents trivial ES. VLSM, very low-speed movement; LSR, low-speed running; MSR, moderate-speed running; HSR, high-speed running; VHSR, very high-speed running.

The relative distance across the quarters of match play in the NHL and AIC is presented in Figure 1. There were no differences in relative distance in the first (*p* = 0.304, ES = 0.17), second (*p* = 0.694, ES = 0.07), third (*p* = 0.968, ES = 0.01), or fourth quarter (*p* = 0.151, ES = 0.24) between the NHL and AIC. The relative distance in each movement category during each quarter of the NHL and AIC is presented in Table 3. Only LSR in the third (*p* = 0.012, ES = 0.38) and fourth quarter (*p* = 0.009, ES = 0.38) was significantly different between the NHL and AIC. No significant differences in the peak running speed were found between the quarters of match play in the NHL (ES = −0.03 to 0.09) or AIC (ES = −0.14 to 0.14). The ES with 95% confidence intervals (CI) for the relative distance in each movement category between the four quarters of the NHL and AIC are presented in Figure 2 and Figure 3, respectively.

The mean game duration during Division 1A+1B, Division 2A+2B, and Division 3A+3B games was 77.0 ± 2.0 min, 76.5 ± 2.4 min, and 76.4 ± 2.2 min, during which referees covered a relative distance of 122.2 ± 10.2 m·min^−1^, 118.4 ± 7.6 m·min^−1^, and 119.9 ± 9.0 m·min^−1^, respectively. There were no significant differences in mean game duration (ES = −0.01 to 0.26) or relative distance (ES = −0.17 to 0.26) between the three divisions. The relative distance in each movement category across the full game for each of the three divisions of the NHL is presented in Table 4. VHSR was higher in Division 3A+3B compared to Division 1A+1B (*p* < 0.001, ES = 0.61) and Division 2A+2B (*p* < 0.001, ES = 0.80). Peak running speed was also higher in Division 3A+3B (6.90 ± 0.60 m·s^−1^) compared to Division 2A+2B (6.42 ± 0.41 m·s^−1^) (*p* = 0.012, ES = 0.55), but there was no significant difference between Division 1A+1B (6.61 ± 0.40 m·s^−1^) and Division 2A+2B or Division 3A+3B.

The mean game duration during the Leinster Championship, Munster Championship, and AIS was 76.6 ± 1.7 min, 77.8 ± 2.3 min, and 76.7 ± 1.9 min, during which referees covered a relative distance of 123.3 ± 12.9 m·min^−1^, 122.7 ± 7.0 m·min^−1^, and 122.6 ± 9.3 m·min^−1^, respectively. There were no significant differences in mean game duration (ES = −0.40 to 0.29) or relative distance (ES = 0.04 to 0.16) between the three phases of the AIC. The relative distance in each movement category across the full game for each of the three phases of the AIC is presented in Table 5. Only HSR differed between phases, with higher values in both the Leinster Championship (*p* = 0.014, ES = 0.39) and Munster Championship (*p* = 0.007, ES = 0.41) compared to the AIS. There was no significant difference in peak running speed between the Leinster Championship (6.71 ± 0.32 m·s^−1^), Munster Championship (6.63 ± 0.38 m·s^−1^), or AIS (6.49 ± 0.39 m·s^−1^) (ES = −0.03 to 0.23).

## 4. Discussion

This study, examining the activity profile of elite hurling referees during games in the NHL and AIC and temporal changes between the match halves and quarters, found no significant differences in the total, VLSM, walking, MSR, HSR, and VHSR distance and peak running speed between competitions. The combined HSR and VHSR distance accounted for 14.4% and 14.8% of the total distance during NHL and AIC games, respectively. There were no differences in the total, VLSM, walking, MSR, HSR, and VHSR distance and peak running speed between NHL and AIC games in the first or second halves. The first quarter of games in both the NHL and AIC was identified as the most intense period, with a greater total, MSR, and HSR distance covered compared to any other quarter of match play.

The average distance covered by hurling referees during games in the NHL and AIC is within 0.9% and 0.2% of the distance covered by elite Gaelic football referees during match play of equivalent national league and AIC games, respectively [9]. As previously mentioned, competitions in hurling and Gaelic football are played on identical playing surfaces, with similar game durations and number of players per team. These common factors may explain, at least in part, the comparable distances covered by elite hurling and elite Gaelic football referees despite differences in game format, playing styles, and formations [1], all of which can influence the movement of the referee. The total distance covered by hurling referees is similar to the ~122 m·min^−1^ of soccer referees [3] and considerably higher than the ~82 m·min^−1^ and 83 m·min^−1^ of rugby league and rugby union referees, respectively [23,24].

Direct comparison of the distance covered at high and very high-speed by hurling referees to other IFTS referees is challenging due to the differences in velocity thresholds. While using standardized movement category velocity thresholds would facilitate such comparisons, arbitrary or generic thresholds have limited internal and ecological validity given their lack of specificity to the activity profile and physical fitness levels of referees. This lack of specificity limits the applicability of findings to referee-specific conditioning programs. Movement categories specifically developed for hurling referees were, therefore, prioritized in this study [19]. However, similar to Gaelic football [9], no differences were found in HSR or VHSR distance between the national league competition and the AIC.

The total distance covered by elite hurling referees during match play is greater than that of elite hurling players, who cover an average of ~108 m·min^−1^ [1]. Similar trends have been found in other IFTSs including Gaelic football and soccer [8,9], where the referees also cover a greater relative distance than players, though the differences between elite hurling referees and players are more pronounced. The greater difference between hurling referees and players may be due to the unique playing style in hurling whereby the ball can travel distances of ~100 m in a very short period of time, with minimal player possessions involved in the completion of a score [1]. This unique pattern of play can reduce the requirement for players to transition with the play and reduce the total running distance whereas the referee is required to keep up with play at all times. In contrast to elite hurling referees, the relative distance covered by elite players was found to be higher during games in the AIC compared to the NHL [4]. However, it is important to note that the difference between competitions for elite hurling players was based on data collected from one team, limiting generalizability. Additionally, the statistical model used did not control for the non-independence of observations arising from multiple data files per player, increasing the risk of type 1 error [25].

Although the HSR threshold for referees in this study is marginally lower than the ≥4.72 m·s^−1^ threshold commonly used for elite players, the average HSR distance covered by elite referees is broadly similar to that of elite players, who average 12–18 m·min^−1^ during competitive match play [1]. During the NHL and AIC, elite players cover ~16.5 m·min^−1^ and ~16.8 m·min^−1^ of HSR [4], which is lower than the average HSR distances of 17.1 m·min^−1^ and 18.0 m·min^−1^ covered by elite hurling referees during the NHL and AIC, respectively. As in the present study, the HSR distance performed by elite players between games in the NHL and AIC was also non-significant.

Temporal differences in the activity profile of elite hurling referees were found during games in both the NHL and AIC, with referees covering a greater total, LSR, MSR, and HSR distance and a lower VLSM and walking distance in the first half compared to the second half. As with elite Gaelic football referees [2], these differences are largely attributed to the higher intensity in the first quarter of games, during which referees covered a greater total, MSR, and HSR distance and less walking distance than in any other quarter of match play. A similar pattern has also been reported for soccer and rugby referees [23,26] and is linked to the increased activity of players during the opening period of play [27].

Examination of the activity profile of elite hurling referees across divisions in the NHL found that referees performed a greater volume of VHSR during games in Division 3A+3B compared to all other divisions. Referees also performed a greater volume of HSR during games in Division 3A+3B, though this was not significant. In the AIC, referees completed significantly less HSR during games in the AIS compared to games in both the Leinster and Munster Championships. No studies have examined differences in the activity profile of elite players between divisions of the NHL or across the phases of the AIC, making it difficult to discern the extent to which player activity influenced these differences. The increased high-speed activity during games in the lower divisions, and the reduced activity during the latter stages of the AIC, may reflect differences in playing styles and tactical approaches. Nonetheless, these differences are important for the physical preparation of elite hurling referees across all phases of both competitions, with further research needed to understand the contributing factors.

Given the similarities in the activity profile of elite hurling referees between games in the NHL and AIC, the level of physical fitness required to officiate and the physical conditioning programs should not, at a fundamental level, differ considerably. However, special consideration must be given to the preparation of referees officiating in the latter stages of the AIC, which take place during the summer when ambient temperatures are higher. The effects of elevated ambient temperatures on physiological function can be considerable, as summer temperatures in Ireland can fluctuate by 15–20 °C in a very short period, providing little to no time for acclimatization. This increased physiological stress can impact both physical and cognitive performance [28,29]. Exercise in the heat can place additional physiological stress on referees, including an elevated heart rate, reduced blood flow to exercising muscles, increased sweat rate, and dehydration [28,29]. Games in the AIC may also require extra time in the event of a draw, with an activity profile similar to the first quarter of match play. Consequently, referees selected to officiate games in the AIC should possess a higher level of physical fitness in response to possible increases in physical exertion.

## 5. Conclusions

This study provides a comprehensive analysis of the activity profile of elite hurling referees during competitive match play in both the NHL and AIC. The total distance, HSR distance, and VHSR distance were consistent between games in the NHL and AIC, with similar activity profiles found across the divisions of the NHL and phases of the AIC. Temporal variations in the total and HSR distance were found between halves, but these differences were primarily attributed to the first quarter of match play, which involved the greatest total and HSR distance compared to all other quarters. These findings can inform the development of conditioning programs that are reflective of the demands of match play. Future research should explore the factors influencing the activity profile of elite hurling referees, evaluate the efficacy of various training methods in enhancing the physical fitness levels required for officiating at the elite level, and examine how physical fitness level impacts the decision-making accuracy of elite IFTS referees.

## Figures and Tables

**Table 1:** Distance in each movement category during match play in the NHL and AIC.

	NHL	AIC	*p*	ES (95% CI of ES)
VLSM (m·min^−1^)	2.1 ± 0.5	1.8 ± 0.4	0.336	0.13 (−0.16 to 0.41)
Walking (m·min^−1^)	24.3 ± 3.3	24.3 ± 3.6	0.743	0.04 (−0.24 to 0.33)
LSR (m·min^−1^)	30.3 ± 3.8	32.1 ± 4.5	0.044	−0.24 (−0.52 to 0.05)
MSR (m·min^−1^)	46.7 ± 6.2	46.4 ± 7.2	0.254	0.15 (−0.14 to 0.43)
HSR (m·min^−1^)	17.1 ±6.5	18.0 ±7.2	0.500	0.10 (−0.19 to 0.38)
VHSR (m·min^−1^)	0.3 ± 0.4	0.2 ± 0.2	0.131	0.18 (−0.10 to 0.47)

Data are presented as mean ± SD. VLSM, very low-speed movement; LSR, low-speed running; MSR, moderate-speed running; HSR, high-speed running; VHSR, very high-speed running.

**Table 2:** Distance in each movement category during the first and second half of the NHL and AIC.

	Competition	First Half	Second Half	*p*	ES (95% CI of ES)
VLSM (m·min^−1^)	NHL	2.0 ± 0.5	2.2 ± 0.5	<0.001	−0.27 (−0.54 to 0.00)
	AIC	1.8 ± 0.5	1.9 ± 0.5	<0.001	−0.29 (−0.59 to 0.01)
Walking (m·min^−1^)	NHL	23.9 ± 3.6	24.6 ± 3.3	0.023	−0.13 (−0.40 to 0.14)
	AIC	23.7 ± 4.0	24.9 ± 3.7	<0.001	−0.26 (−0.56 to 0.04)
LSR (m·min^−1^)	NHL	31.3 ± 4.0	29.4 ± 4.4	<0.001	0.34 (0.07 to 0.61)
	AIC	32.8 ± 4.8	31.3 ± 4.5	<0.001	0.26 (−0.04 to 0.56)
MSR (m·min^−1^)	NHL	48.4 ± 7.1	45.1 ± 6.5	<0.001	0.38 (0.11 to 0.65)
	AIC	48.1 ± 8.0	44.9 ± 7.6	<0.001	0.38 (0.07 to 0.68)
HSR (m·min^−1^)	NHL	18.2 ± 7.1	16.0 ± 6.7	<0.001	0.31 (0.04 to 0.58)
	AIC	19.4 ± 8.1	16.6 ± 7.1	<0.001	0.32 (0.01 to 0.62)
VHSR (m·min^−1^)	NHL	0.2 ± 0.5	0.3 ± 0.5	0.831	−0.02 (−0.29 to 0.25)
	AIC	0.2 ± 0.3	0.2 ± 0.4	0.623	−0.04 (−0.34 to 0.26)

Data are presented as mean ± SD. VLSM, very low-speed movement; LSR, low-speed running; MSR, moderate-speed running; HSR, high-speed running; VHSR, very high-speed running.

**Table 3:** Distance in each movement category across the four quarters of match play in the NHL and AIC.

	Competition	First Quarter	Second Quarter	Third Quarter	Fourth Quarter
VLSM (m·min^−1^)	NHL	1.9 ± 0.6	2.1 ± 0.6 ^b^	2.2 ± 0.6 ^a^	2.2 ± 0.6 ^a^
	AIC	1.7 ± 0.6	1.8 ± 0.6	1.9 ± 0.6	1.9 ± 0.5 ^a^
Walking (m·min^−1^)	NHL	22.6 ± 4.0	25.0 ± 4.0 ^a^	23.9 ± 3.6 ^a^	25.2 ± 4.3 ^a,d^
	AIC	22.6 ± 4.2	24.6 ± 4.4 ^a^	24.0 ± 3.6 ^a^	25.7 ± 4.3 ^a,c^
LSR (m·min^−1^)	NHL	32.9 ± 4.7	29.8 ± 5.3 ^a^	30.0 ± 5.2 ^a^	28.4 ± 5.1 ^a^
	AIC	33.5 ± 5.2	32.2 ± 5.9	32.2 ± 5.5 ^†^	31.0 ± 5.1 ^a,†^
MSR (m·min^−1^)	NHL	52.1 ± 7.9	45.2 ± 8.8 ^a^	46.6 ± 7.3 ^a^	43.6 ± 8.6 ^a,d^
	AIC	51.6 ± 10.3	45.2 ± 9.0 ^a^	46.7 ± 9.4 ^a^	44.5 ± 9.0 ^a^
HSR (m·min^−1^)	NHL	20.0 ± 8.4	16.6 ± 6.9 ^a^	16.5 ± 7.9 ^a^	15.7 ± 8.1 ^a^
	AIC	21.0 ± 9.3	18.1 ± 8.8 ^a^	16.7 ± 8.4 ^a^	17.0 ± 8.1 ^a^
VHSR (m·min^−1^)	NHL	0.2 ± 0.6	0.3 ± 0.7	0.3 ± 0.6	0.2 ± 0.5
	AIC	0.1 ± 0.3	0.2 ± 0.5	0.2 ± 0.4	0.2 ± 0.6

Data are presented as mean ± SD. ^a^ *p* <0.001 vs. first quarter; ^b^ *p* <0.01 vs. first quarter; ^c^ *p* <0.001 vs. third quarter; ^d^ *p* <0.01 vs. third quarter; ^†^ *p* <0.05 vs. NHL. VLSM, very low-speed movement; LSR, low-speed running; MSR, moderate-speed running; HSR, high-speed running; VHSR, very high-speed running.

**Table 4:** Distance in each movement category during the full game in the three divisions of the NHL.

	Division 1A+1B	Division 2A+2B	Division 3A+3B
VLSM (m·min^−1^)	2.0 ± 0.4	2.1 ± 0.5	2.2 ± 0.5
Walking (m·min^−1^)	23.5 ± 3.1	25.5 ± 3.2	24.5 ± 3.5
LSR (m·min^−1^)	31.7 ± 3.1	29.0 ± 4.0	27.6 ± 3.9
MSR (m·min^−1^)	47.1 ± 6.6	46.2 ± 5.6	46.3 ± 6.5
HSR (m·min^−1^)	17.7 ± 7.3	15.4 ± 5.1	18.6 ± 5.7
VHSR (m·min^−1^)	0.2 ± 0.3	0.1 ± 0.3	0.7 ± 0.8 ^a^

Data are presented as mean ± SD. ^a^ *p* <0.01 vs. All other phases. VLSM, very low-speed movement; LSR, low-speed running; MSR, moderate-speed running; HSR, high-speed running; VHSR, very high-speed running.

**Table 5:** Distance in each movement category during the full game in the three phases of the AIC.

	Leinster	Munster	All-Ireland Series
VLSM (m·min^−1^)	1.9 ± 0.5	1.8 ± 0.4	1.8 ± 0.4
Walking (m·min^−1^)	23.6 ± 3.5	24.6 ± 3.7	24.7 ± 3.8
LSR (m·min^−1^)	31.2 ± 3.4	31.9 ± 4.8	32.9 ± 4.9
MSR (m·min^−1^)	45.3 ± 7.5	46.2 ± 6.1	47.5 ± 7.8
HSR (m·min^−1^)	21.1 ± 8.5	17.9 ± 5.4	15.7 ± 6.7 ^a^
VHSR (m·min^−1^)	0.2 ± 0.2	0.2 ± 0.3	0.1 ± 0.2

Data are presented as mean ± SD. ^a^ *p* <0.05 vs. all other phases. VLSM, very low-speed movement; LSR, low-speed running; MSR, moderate-speed running; HSR, high-speed running; VHSR, very high-speed running.

## Data Availability

The raw data supporting the conclusions of this article will be made available by the authors on request.
